# Adult global deletion of C1q reduces reactive astrocytes and rescues synaptic loss independently of amyloid phagocytosis in AD mouse model

**DOI:** 10.1002/alz70855_104516

**Published:** 2025-12-24

**Authors:** Tiffany J Petrisko, Angela Gomez‐Arboledas, Shu‐Hui Chu, Andrea J Tenner

**Affiliations:** ^1^ University of California, Irvine, Irvine, CA, USA

## Abstract

**Background:**

The complement system contributes to enhanced inflammation and cognitive decline in Alzheimer's disease (AD). Previous studies have demonstrated constitutive deletion of the classical initiator protein, C1q, reduces glial activity and attenuates neuronal loss in AD mouse models. The objective of this study was to determine if global deletion of C1q at two different stages of Alzheimer's disease would reduce neuroinflammation, synaptic loss, and amyloid engulfment.

**Method:**

Briefly, C1qa^FL/FL^RosaCre^ERT2^ mice were crossed to the Arctic AD mouse model to generate WT and Arctic (Arc) C1qa^FL/FL^ mice with and without the RosaCre^ERT2^ transgene. Mice were treated with tamoxifen at either 11‐wks or 20‐wks of age to induce global C1q deletion. Brains were collected at 10m of age and stained for C3/GFAP and C5aR1/Iba1 to characterize neuroinflammation. Superresolution microscopy was utilized to assess synaptic density of Vglut1‐Psd95 synapses while confocal microscopy was used to quantify the phagocytosis of amyloid by microglia.

**Result:**

Early deletion of C1q at 11‐wks of age failed to reduce microglial reactivity as measured by C5aR1 or Iba1 hippocampal volume while deletion at 20‐wks significantly reduced Iba1 volume (29%) alongside a trending reduction in C5aR1 expression (32%). Deletion of C1q at 11‐wks of age induced a trending reduction in GFAP volume (25%) while significantly reducing C3 expression (48%). In contrast, deletion at 20‐wks failed to reduce GFAP reactivity but a trending reduction of C3 expression was observed (33%). At 10m of age, Arc mice displayed reduced colocalization of Vglut1‐Psd95 synapses in the CA3 region of the hippocampus. Deletion of C1q at either 11‐ or 20‐ wks of age rescued synaptic loss. Arc mice with C1q deleted at 11‐wks displayed increased microglial (Iba1) colocalization with amyloid (6E10) but no changes in lysosome associated phagocytosis of amyloid was observed at either timepoint.

**Conclusion:**

The data presented here demonstrates that global deletion of C1q in the Arctic mouse model results in reduced expression of C3 and GFAP in hippocampal astrocytes. Additionally, global C1q deletion rescues synaptic density regardless of the age of deletion independent of amyloid phagocytosis.

